# New Ascomycetes from the Mexican Tropical Montane Cloud Forest

**DOI:** 10.3390/jof9090933

**Published:** 2023-09-15

**Authors:** Tania Raymundo, Ricardo Valenzuela, César Ramiro Martínez-González, Jesús García-Jiménez, Aurora Cobos-Villagrán, Marcos Sánchez-Flores, Javier de la Fuente, Michelle Martínez-Pineda, Abigail Pérez-Valdespino, Julio Cesar Ramírez-Martínez, Isolda Luna-Vega

**Affiliations:** 1 Instituto Politécnico Nacional, Escuela Nacional de Ciencias Biológicas, Departamento de Botánica, Laboratorio de Micología, Mexico City 11340, Mexico; traymundoo@ipn.mx (T.R.); rvalenzg@ipn.mx (R.V.); cobos.fungi@gmail.com (A.C.-V.); ing.michellemsp@gmail.com (M.M.-P.); 2 Departamento de Fitotecnia, Instituto de Horticultura, Universidad Autónoma Chapingo, km 38.5 Carretera Federal México-Texcoco, Texcoco 56230, Estado de México, Mexico; ramiro_mg.unam@ciencias.unam.mx; 3 Instituto Tecnológico de Ciudad Victoria, Tecnológico Nacional de México, Blvd. Emilio Portes Gil #1301Pte, Ciudad Victoria 87010, Tamaulipas, Mexico; jgarjim@yahoo.com.mx (J.G.-J.); sanflores37@gmail.com (M.S.-F.); 4 Colegio de Posgraduados, km 36.5, Montecillo, Texcoco 56230, Estado de México, Mexico; jdelafuenteitcv@gmail.com; 5 Instituto Politécnico Nacional, Escuela Nacional de Ciencias Biológicas, Departamento de Bioquímica, Laboratorio de Ingeniería Genética, Mexico City 11340, Mexico; abperezv@ipn.mx; 6 Laboratorio de Biogeografía y Sistemática, Departamento de Biología Evolutiva, Facultad de Ciencias, Universidad Nacional Autónoma de México, Mexico City 04510, Mexico; rasec.jc@ciencias.unam.mx

**Keywords:** Ascomycota, new species, phylogeny, taxonomy, biogeography

## Abstract

The tropical montane cloud forest is the most diverse and threatened vegetation type in Mexico. In the last decade, the number of described Ascomycetes species has notably increased, reaching more than 1300 species. This study describes six new species based on their molecular and morphological characteristics. Our results suggest that Mexico has the highest number of described species in the Neotropics. However, many other Mexican lineages still need to be described.

## 1. Introduction

The fungi of the phylum Ascomycota, such as endophytes, mycorrhiza, phytopathogens, and saprobes, have various symbiotic ecological functions, with the latter producing many enzymes that degrade complex polymers such as starch, cellulose, chitin, keratin, and lignin [1]. These functions serve to balance the ecosystem. In tropical regions, species diversity is due to the structural complexity of microclimates and microhabitats. In this sense, the Mexican tropical montane cloud forest (TMCF) has been cataloged as the most diverse per area unit [2,3], which is also reflected by the Ascomycetes group; a significant number of species of this group have been described recently.

Mexico comprises a wide variety of vegetation types. One of the most diverse ecosystems, which is under significant threat, is the TMCF, also known as bosque mesófilo de montaña or bosque nuboso (cloud forest), which is characterized mainly by the presence of clouds at the vegetation level. The TMCF is characterized by high levels of atmospheric humidity, 1500–3000 mm of rainfall, and temperatures of 12–23 °C. The vegetation types in this ecosystem develop in rugged reliefs with a discontinuous distribution pattern, analogous to an archipelago of islands, and in ravines or slopes in the Sierra Madre Occidental to the north of Sinaloa, Nayarit, Jalisco, Colima, and Michoacán; in the Sierra Madre Oriental, from southwestern Tamaulipas to northern Oaxaca, including portions of San Luis Potosí, Hidalgo, Puebla, and Veracruz; and in the Sierra Madre south of Guerrero and Oaxaca. In addition, TMCF is also located in some areas of the Trans-Mexican Volcanic Belt. The flora has geographical links with North America in the tree layer and with South America in the herbaceous and shrub layers; it is closely related to Asian flora. In Mexico, these forests are vital due to their extraordinary biodiversity. Between 2500 and 3000 species of vascular plants inhabit the TMCF of Mexico, representing approximately 10% of its floristic richness, making it the country’s most diverse per area unit [1]. According to Guzmán [4], exhaustive monographic studies of 22 genera of Ascomycota are available.

In 2008, the existence of 1335 species of Ascomycetes was reported in Mexico [5]. These species reportedly belong to 41 orders, 126 families, and 441 genera, including 35% of lichenized ascomycetes and 4.9% marine taxa, without considering the asexual phases. In the same year, Heredia-Abarca et al. [6] registered 1353 anamorph species. Subsequently, Aguirre-Acosta et al. [7] noted that the CONABIO catalog by Cifuentes [8] enlisted 646 species of Ascomycota in Mexico without considering the asexual phases, distributed in 86 families and 275 genera, including lichens. Later, Del Olmo et al. [9] noted that in Mexico, there are 954 Ascomycota species in the TMCF. According to the authors, these fungi are assigned to 10 taxonomic classes: Arthoniomycetes (10 species), Dothideomycetes (125), Eurotiomycetes (35), Geoglossomycetes (2), Lecanoromycetes (167), Leotiomycetes (66), Orbiliomycetes (3), Pezizomycetes (93), Saccharomycetes (1), and Sordariomycetes (333), with 119 incertae sedis.

The main objective of this study was to contribute to the cataloguing of new species of Ascomycetes in the TMCF and update the knowledge on the Ascomycetes richness in this threatened ecosystem type. We aimed to describe, phylogenetically and morphologically, six Ascomycetes species distributed in the Mexican tropical montane cloud forest, an ecosystem in danger of extinction.

## 2. Material and Methods

### 2.1. Morphological Studies

Specimens from the “Dr. Gastón Guzmán Huerta” fungal collection at the Herbarium of the Escuela Nacional de Ciencias Biológicas, Instituto Politécnico Nacional, Mexico City, Mexico (ENCB), and the “Jose Castillo Tovar” collection at the Instituto Tecnológico de Ciudad Victoria (ITCV) were revised. The color codes follow Kornerup and Wanscher [10] and Bessette et al. [11]. Microscopic observations were made of tissues rehydrated in 5% aqueous KOH and Melzer’s reagent; ascospore dimensions included the ornamentation. The macroscopic features were photographed with a Nikon D7000 camera and the micrographs with a Sony DSCWX350. Additionally, scanning electron microscopy (SEM; Hitachi SU1510, Hitachi, Tokyo, Japan) was used to observe the details of spore walls. The meanings of the taxonomic terms are based on Ulloa and Hanlin [12].

### 2.2. Amplification and Sequencing

DNA was obtained from herborized exemplars. Genomic DNA was extracted using the CTAB method [13]. The DNA was quantified with a NanoDrop 2000c (Thermo, Waltham, MA, USA). Dilutions were prepared from each sample at 20 ng/µL to amplify 4 regions: internal transcribed spacer rDNA-ITS1 5.8S rDNA-ITS2 (ITS), large nuclear subunit ribosomal DNA (nLSU), the second largest subunit of the RNA polymerase II gene (*rpb2*), and the region of the small mitochondrial subunit (mtSSU). The reaction mixture for PCR was prepared at a final volume of 15 µL and contained 1× buffer, 0.8 mM dNTPs mix, 20 pmol of each primer, 2 units of GoTaq DNA (Promega, Madison, WI, USA), and 100 ng of template DNA. The PCR products were verified by agarose gel electrophoresis. The gels were run for 1 h at 95 V cm^−3^ in 1.5% agarose and 1× Tris acetate-EDTA (TAE) buffer. The gels were stained with GelRed (Biotium, Fremont, CA USA), and the bands were visualized in an Infinity 3000 transilluminator (Vilber Lourmat, Eberhardzell, Germany). The amplified products were purified with an ExoSAP purification kit (Affymetrix, Santa Clara, CA, USA), following the manufacturer’s instructions. They were quantified and prepared for sequence reaction using a BigDye Terminator v.3.1 (Applied Biosystems, Foster City, CA, USA). These products were sequenced in both directions with an Applied Biosystems 3730XL DNA analyzer (Applied Biosystems, Foster City, CA, USA) at the Instituto de Biología of the Universidad Nacional Autónoma de México (UNAM). The sequences obtained were compared with the original chromatograms to detect and correct possible reading errors. The sequences of both strands of each gene were analyzed, edited, and assembled using BioEdit v. 7.0.5 [14] to generate a consensus sequence, which was compared with those deposited in GenBank [15] using BLASTN v. 2.2.9 [16].

### 2.3. Phylogenetic Analysis

Alignment was carried out based on the taxonomic sampling method employed by Pem et al. [17] to explore the phylogenetic relationships of the new species of *Holmiella* (Table 1). Each gene region was independently aligned using the online version of MAFFT v. 7 [18,19,20]. The alignment was reviewed in PhyDE v.10.0 [21], followed by minor manual adjustments to ensure character homology between taxa. A matrix was formed for ITS with 10 taxa (690 characters) for ITS, 23 taxa (831 characters) for LSU, and 14 taxa (640 characters) for mtSSU. The aligned matrices were concatenated into a single matrix (24 taxa, 2161 characters). Three partitioning schemes were established, one each for the ITS, nLSU, and mtSSU, using the option to minimize the stop codon with Mesquite v3.70 [22].

Alignment was carried out based on the taxonomic sampling method employed by Sun et al. [23] to explore the phylogenetic relationships of the new species of *Kirschsteiniothelia* (Table 2). Each gene region was independently aligned using the online version of MAFFT v. 7 [18,19,20]. The alignment was reviewed in PhyDE v.10.0 [21], followed by minor manual adjustments to ensure character homology between taxa. A matrix was formed with 23 taxa (695 characters) for ITS and 37 taxa (836 characters) for LSU. The aligned matrices were concatenated into a single matrix (37 taxa, 1534 characters). Two partitioning schemes were established, one each for the ITS and LSU, using the option to minimize the stop codon with Mesquite v3.70 [22].

Alignment was carried out based on the taxonomic sampling method employed by Healy et al. [24] to explore the phylogenetic relationships of the new species of *Microglossum* (Table 3). Each gene region was independently aligned using the online version of MAFFT v. 7 [18,19,20]. The alignment was reviewed in PhyDE v.10.0 [21], followed by minor manual adjustments to ensure character homology between taxa. A matrix was formed with 61 taxa (690 characters) for ITS, 23 taxa (831 characters) for LSU, and 22 taxa (670 characters) for the second largest subunit of the RNA polymerase II gene (*rpb2*). The aligned matrices were concatenated into a single matrix (61 taxa, 2191 characters). Five partitioning schemes were established, one each for the ITS and nLSU and three for the *rpb2* gene region, using the option to minimize the stop codon with Mesquite v3.70 [22].

Alignment was carried out based on the taxonomic sampling method employed by [25] to explore the phylogenetic relationships of the new species of *Claussenomyces* (Table 4). First, the ITS region was aligned using the online version of MAFFT v. 7 [18,19,20]. Next, the alignment was reviewed in PhyDE v.10.0 [21], followed by minor manual adjustments to ensure character homology between taxa. The matrix was composed of 22 taxa (700 characters).

Alignment was carried out based on the taxonomic sampling method employed by Argnello et al. [26] and Healy et al. [24] to explore the phylogenetic relationships of the new species of *Wolfina* (Table 5). The ITS region was aligned using the online version of MAFFT v.7 [18,19,20]. The alignment was reviewed in PhyDE v.10.0 [21], followed by minor manual adjustments to ensure character homology between taxa. The matrix was composed of 11 taxa (700 characters).

Alignment was carried out to resolve the phylogenetic relationships of the new species of *Dematophora* based on the taxonomic sampling method employed by Wittstein et al. [27] (Table 6). Each gene region was independently aligned using the online version of MAFFT v. 7 [18,19,20]. The alignment was reviewed in PhyDE v.10.0 [21], followed by minor manual adjustments to ensure character homology between taxa. A matrix was formed with 30 taxa (699 characters) for ITS and 18 taxa (836 characters) for LSU. The aligned matrices were concatenated into a single matrix (30 taxa, 1535 characters). Two partitioning schemes were established, one each for the ITS and LSU, using the option to minimize the stop codon with Mesquite v3.70 [22].

The region was aligned independently using the online version of MAFFT v7 [18,19,20]. The alignments were reviewed in PhyDE [21], followed by minor manual adjustments to maximize character similarity. Phylogenetic inferences were estimated with maximum likelihood in RAxML v. 8.2.10 [28] with a GTR + G model of nucleotide substitution. We ran 1000 rapid bootstrap replicates with the GTRGAMMA model to assess branch support. For Bayesian posterior probability, the best evolutionary model for alignment was sought using PartitionFinder v.2.0 [29,30,31]. Phylogenetic analyses were performed using MrBayes v. 3.2.6 x64 [32]. The information block for matrices included two simultaneous runs, four Monte Carlo chains, temperature set to 0.2 °C, and sampling of 10 million generations (standard deviation ≤0.1) with trees sampled every 1000 generations. The first 25% of samples were discarded as burn-in, and stationarity was checked in Tracer v. 1 [33]. Finally, the trees were visualized and optimized in FigTree v. 1.4.4 [34] and edited in Adobe Illustrator vCS4 (Adobe Systems, Inc., San Jose, CA, USA).

## 3. Results

### 3.1. Taxonomy

#### 3.1.1. *Dothideomycetes*, *Patellariales*, *Patellariaceae*

1. ***Holmiella hidalgoensis*** Raymundo, Martínez-González & R. Valenz. sp. nov.

**MycoBank:** MB842041.

**Figure 1:**
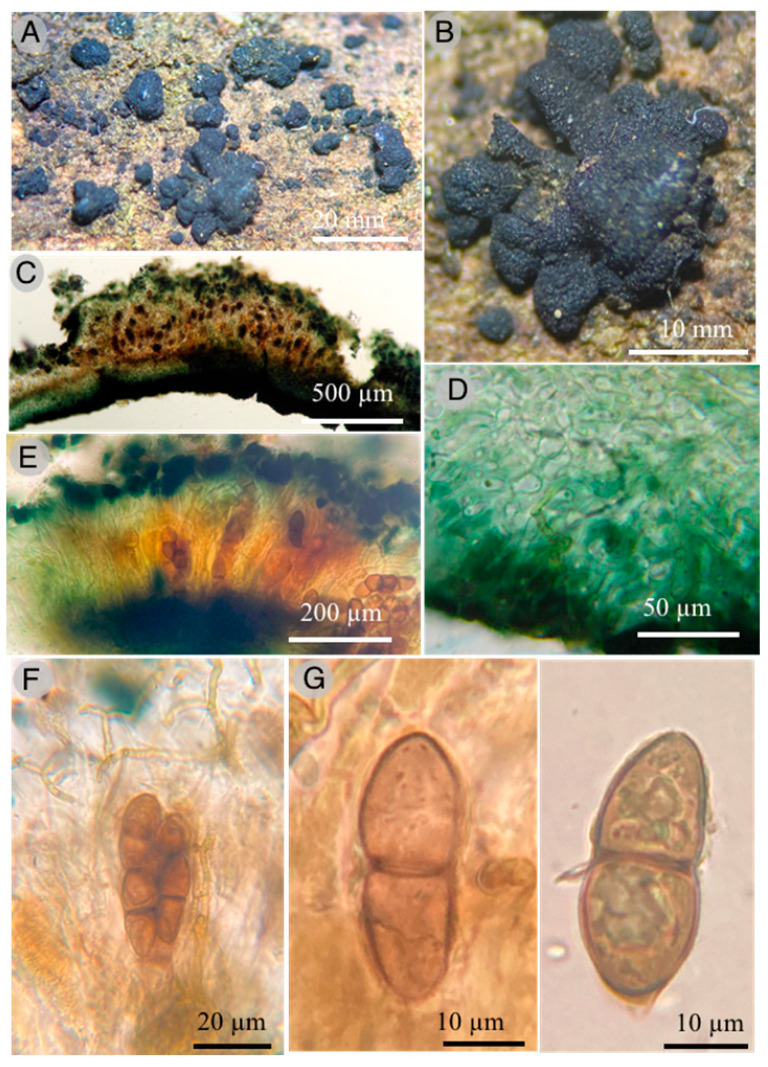
*Holmiella hidalgoensis* T. Raymundo 4608 Holotipe (**A**,**B**) Ascomata; (**C**) optical microscope images through the ascoma; (**D**) microscope image of ectal excipulum; (**E**) optical microscope images of hymenium; (**F**) optical microscope images of asci with ascospores; (**G**) optical microscope images of ascospores.

**Figure 2:**
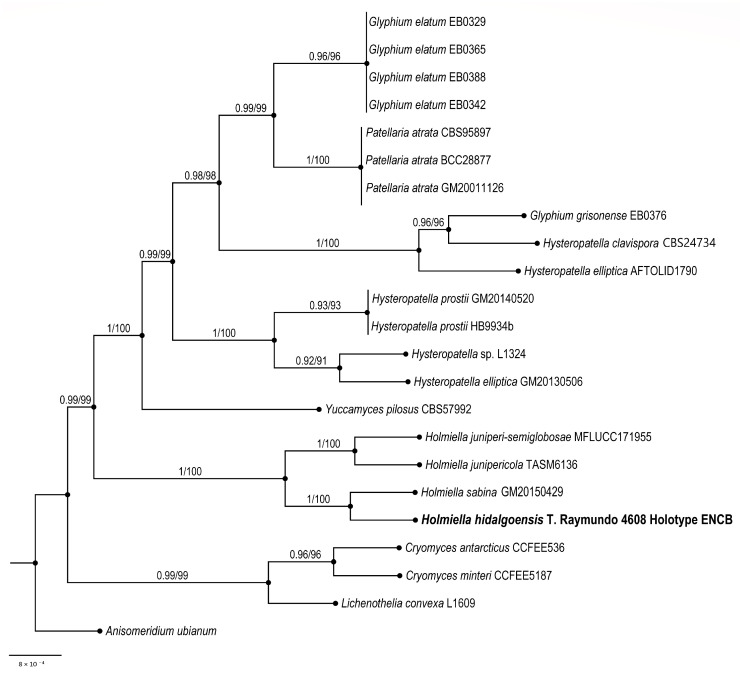
Bayesian inference phylogram of ITS, LSU, and SSU sequence data. Posterior probability (left of slash) from Bayesian analysis and bootstrap support (right of slash) are given above the nodes. New species *Holmiella hidalgoensis* is shown in bold.

**Figures:** Figure 1 and Figure 2.

**Diagnosis:** Ascomata discoidal to ovoid, black, 1–1.5 × 600–800 µm; asci hyaline, pedicellate, bitunicate, 40–45 × 12–16 µm; ascospores ellipsoid to fusoid, golden brown, transverse septae, uniseriate to irregular biseriate, 32–36 × 10–12 µm.

**Type:** MEXICO: Hidalgo, Zacualtipán de Ángeles municipality, Bosque El Hayal, sobre la desviación a Tlahuelompa, 20°37′34′’ N, 98°37′07′’ W, 2250 m, 2 July 2013, T. Raymundo 4608 (ENCB).

**GenBank:** ITS OQ877252, nrLSU OQ880481, SSU OQ878242.

**Etymology:** The epithet indicates that the species grows in Hidalgo.

Ascomata 1–1.5 mm diameter, 600–800 µm thick, solitary to gregarious, erumpent to superficial, rounded to angular, discoidal to powdery, 1 to 1.5 mm diameter, sessile, rough, exposing the asci, black color; basal peridium green to black, 160-200 µm thick; paraphysoids 3–3.5 µm in diameter, filiform, branched, anastomosed, deep green; paraphyses protruding from asci; asci 40–45 × 12–16 µm, bitunicate, cylindrical to clavate, sessile, rounded at the apex, octosporate; ascospores uniseriate to irregularly biseriate in the apical part (28.8–) 32–36 (40) × (9.6–) 10–12 (–12.8) µm broadly ellipsoid to fusiform, bicellular, with one septum and constricted in the middle part, the distal portion slightly larger than the proximal, pale yellowish to dark golden brown when ripe.

**Habitat:** Gregarious on decaying branches of angiosperms.

**Additional specimens examined:** MEXICO: Hidalgo, Zacualtipán de Ángeles municipality, Bosque El Hayal, sobre la desviación a Tlahuelompa, 20°37′34″ N, 98°37′07″ W, 2250 m, 2 July 2013, R. Valenzuela 14997 (ENCB, Paratype).

**Taxonomical notes:** This species is characterized by pulvinated ascomata, black and erumpent, ascospores 32–36 × 10–12 µm, bicellular, with a golden-brown color and a germinative pore. Morphologically and phylogenetically, it is close to *Holmiella sabina* (De Not.) Petrini, Samuels & E. Müll. However, the former presents ascomata with toothed margins, ascospores of 25–40 × 13–20 µm, bicellular, reddish-brown with two germinative pores. *Holmiella juniperi-semiglobosae* Pem, Gafforov, Jeewon & K.D. Hyde and *H. junipericola* Pem, Gafforov, Jeewon & K.D. Hyde are species that are phylogenetically related; however, they grow on *Juniperus semiglobosa* and *J. zerawschanica,* respectively, from Uzbekistan [17].

#### 3.1.2. Pleosporales, Kirschteiniotheliaceae

2. ***Kirschsteiniothelia esperanzae*** Raymundo, Cobos-Villagrán & R. Valenz. sp. nov.

**MycoBank:** MB822042.

**Figure 3:**
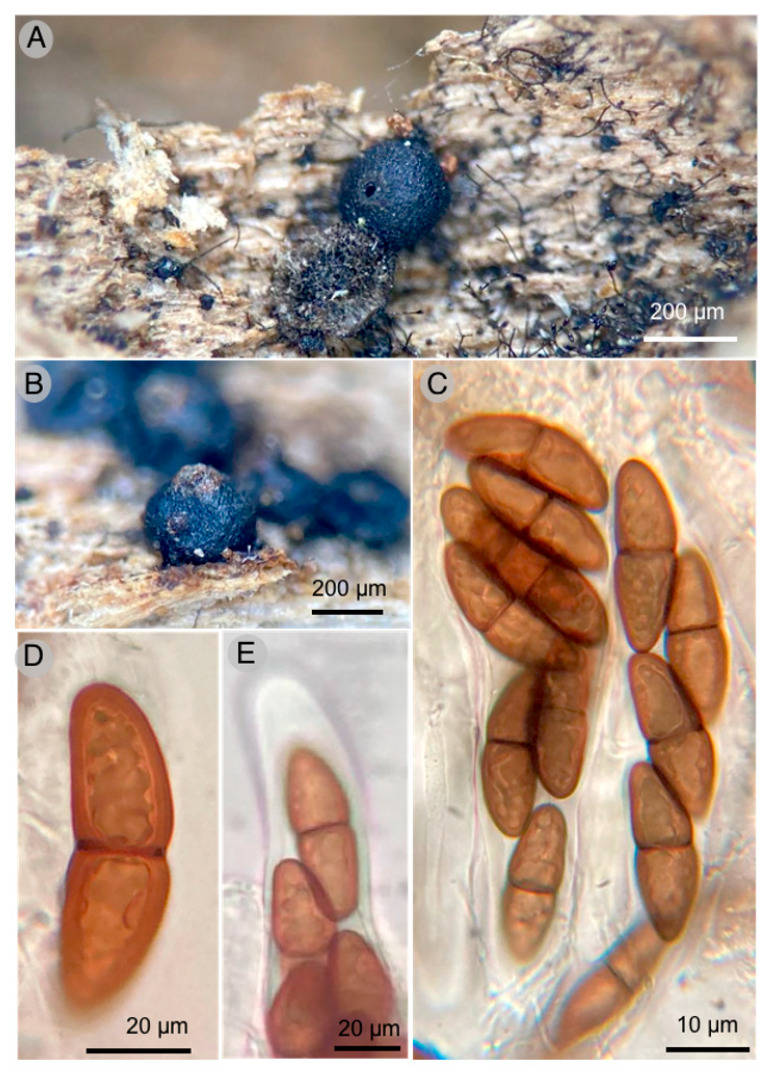
*Kirschsteiniothelia esperanzae* T. Raymundo 6581 Holotype: (**A**) pseudothecia showing ostiole; (**B**) pseudothecia; (**C**) optical microscope images of asci with ascospores; (**D**) optical microscope images of ascospore; (**E**) apical part of asca.

**Figure 4:**
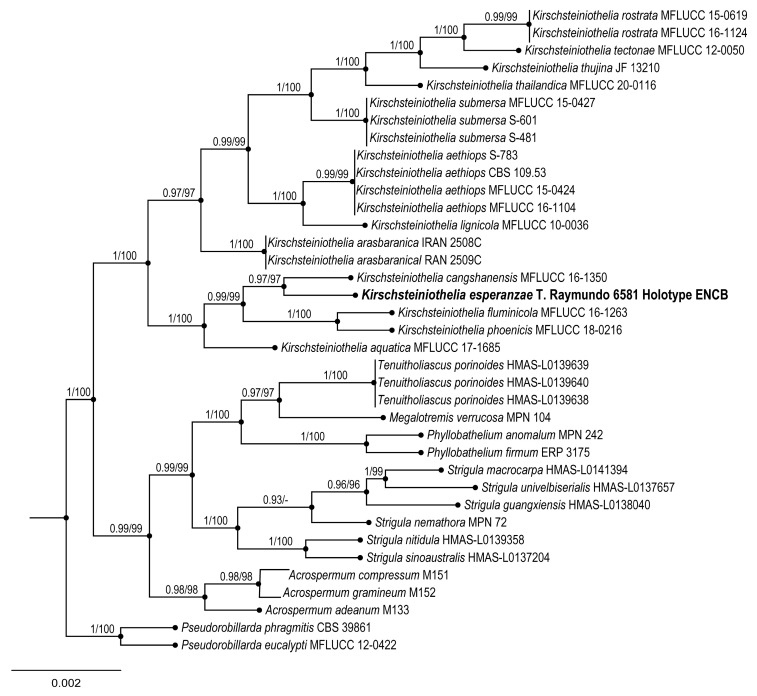
Bayesian inference phylogram of ITS, LSU, and *rpb*2 sequence data. Posterior probability (left of slash) and bootstrap support values (right of slash) in Bayesian analysis are given above the nodes. New species *Kirschsteiniothelia esperanzae* is shown in bold.

**Figures:** Figure 3 and Figure 4.

**Diagnosis:** Ascomata 300–700 µm diameter × 600–700 µm high, peridium 55 to 100 μm thick, pseudoperiphyses 5 μm wide, asci (168–)178–203 × 32–35 μm and ascospores 40–50(–53) × 14–17 μm.

**Type:** MEXICO: Oaxaca, Sierra de Juárez, Ixtlán district, Santiago Comaltepec, La Esperanza, Carretera Oaxaca-Tuxtepec km 51, 17°37′55″ N, 96°22′01″ W, 1600 m, 21 May 2017; T. Raymundo 6581 (ENCB, Holotype).

**GenBank:** ITS: OQ877253, LSU: OQ880482.

**Etymology:** The epithet refers to La Esperanza’s locality, where the species was collected.

Pseudothecia 400–700 µm diameter × 600–700 µm high, hemispherical to globose-subglobose, generally aggregated, seldom dispersed, completely superficial when mature, black, with a well-defined brown ostiole; peridium 55–100 µm thick, tapering at the base, 55 µm, laterally to 60 µm and broader at the base of the ostiole, up to 100 µm wide, pseudoparenchymatous, composed of isoradiated cells of (10–) 16–20 × (11–) 12–15 µm, prismatic texture, slightly thick walls, 1–1.5 µm; pseudoparaphysis very thick up to 5 µm wide, hyaline, branched and anastomosed; asci (168–) 178–203 × 32–35 µm, bitunicate, fisitunicate, fusiform to soleiform, with internal apical beak, eight spores; ascospores 40–50 (–53) × 14–17 µm, ellipsoid or soleiform, 1-septate, slightly constricted at the septum, light brown to olive-brown, smooth.

**Habitat:** Gregarious on decaying wood.

**Taxonomical notes:** This species is characterized by having larger asci (168–)178–203 × 32–35 µm and ascospores 40–50(–53) × 14–17 µm. This species is morphologically similar to *Kirschsteiniothelia thujina* (Peck) D. Hawksw. due to the long ascomata (300–600 µm) and ascospores. However, *K. thujina* has a dark reddish-brown ostiole and an angular texture in the peridium, and the host is *Abies balsamea* Mill. and *Thuja occidentalis* L. *Kirschsteiniothelia esperanzae* has a brown ostiole and peridium cells with prismatic texture, and the host is not identified. This last species was collected in the *Oreomunnea mexicana* (Standl.) J.-F.Leroy TMCF of Oaxaca. Phylogenetic data confirm that *K. esperanzae* is a new species, close to *K. thujina* and *K. rostrata* Jing Yang & K.D. Hyde. These two species and *K*. *arasbaranica* Mehrabi, R. Hemmati & Asgari form a large clade. These three species have the largest ascospores of the group, more than 30 × 15 µm [35,36].

#### 3.1.3. Geoglossomycetes, Geoglossales, Geoglossaceae

3. ***Microglossum flavoviride*** Sánchez-Flores, García-Jiménez & Raymundo sp. nov.

**MycoBank:** MB842043.

**Figure 5:**
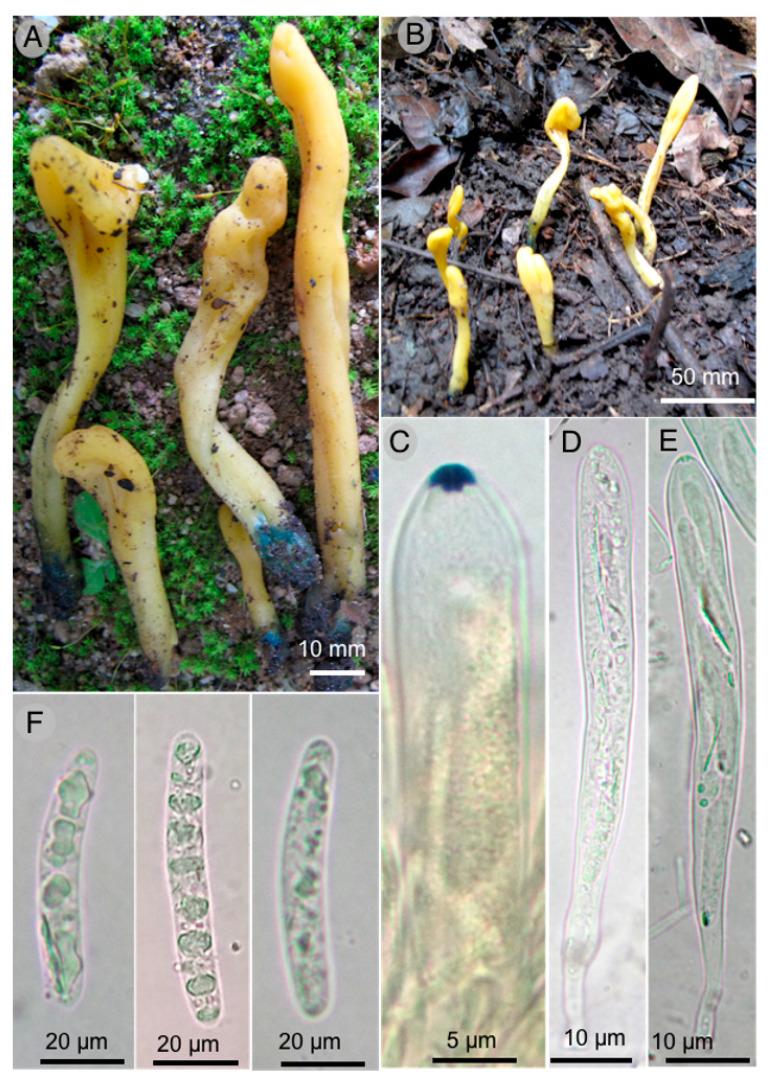
*Microglossum flavoviride* García 18649 Holotype: (**A**,**B**) ascomata; (**C**) amyloid operculum; (**D**) immature ascus; (**E**) mature ascus; (**F**) ascospores.

**Figure 6:**
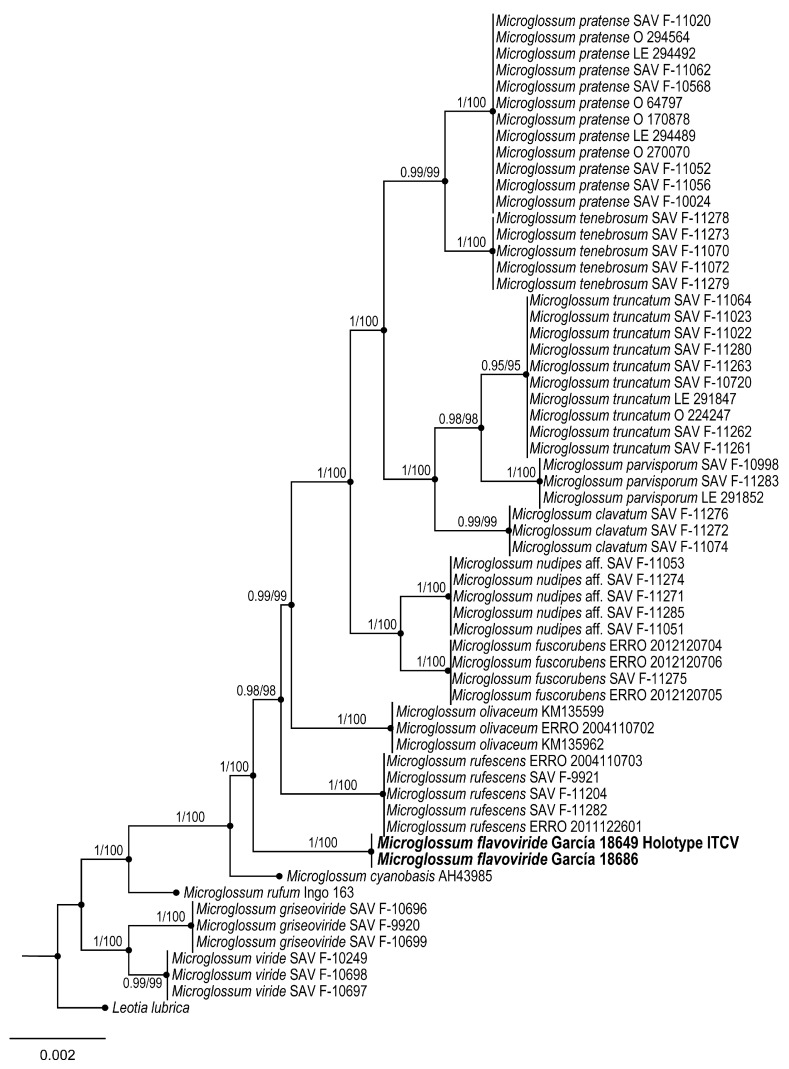
Bayesian inference phylogram of ITS, LSU, and *rpb*2 sequence data. Posterior probability (left of slash) from Bayesian analysis and bootstrap support (right of slash) are given above the nodes. New species *Microglossum flavoviride* is shown in bold.

**Figures:** Figure 5 and Figure 6.

**Diagnosis:** Ascomata 16–65 × 4–9 mm, gregarious, lanceolate to spatulate, yellowish-green and deep green at the base, asci 111–160 × 11–14 µm, octosporate, hyaline, ascospores (20–) 22–45 × 4–6 (–6.5) µm, bacilliform, cylindrical, with 6–10 septa.

**Type:** MEXICO: Chiapas, Ocozocoautla municipality, Laguna Bélgica, 16°52′44.12″ N, 93°27′25.64″ W, 1004 m, 16 August 2011, J. García 18649 (ITCV, Holotype).

GenBank: ITS: OQ877254, LSU: OQ880483.

**Etymology:** It was named flavoviride for the ascoma color.

Ascomata 16–65 mm long, gregarious, lanceolate to spatulate, yellowish-green (30A7) color, deep green (27E8) at the base, cartilaginous consistency, viscous-moist texture; stipe 14–36 mm long, 2 mm wide toward the apex and 4–9 mm wide toward the base, flattened laterally, hollow, turns green when cut, fertile part 10–30 × 4–9 mm; medullar excipulus with intricate texture, formed by hyphae 3–8 µm in diameter, hyaline, indistinguishable subhymen; hymenium 160–185 µm thick; paraphyses 2–4 (–5) µm diameter, filiform, hyaline, septate, bifurcate toward the base, blunt apex, nodulous, irregular to rounded, hook-shaped to straight; asci 111–160 × 11–14 µm, octosporate, hyaline, clavate, amyloid operculum; ascospores (20–) 22–45 × 4–6 (–6.5) µm, bacilliform, cylindrical, slightly allantoic to spindle-shaped, hyaline, multigutulate, 6–10 septa not very visible.

**Additional specimens**: MEXICO. Chiapas, Ocozocoautla municipality, Laguna Bélgica, 16°52′44.12″ N, 93°27′25.64″ W, 1004 m, 16 August 2011, J. García 18686 (ITCV).

**Taxonomical notes:** Ascomata 16–65 mm long, yellowish-green and deep green at the base, ascospores (20–) 22–45 × 4–6 (–6.5) µm, bacilliform, cylindrical, slightly allantoic to spindle-shaped, hyaline, 6-10 septa. It can be confused with *M. rufum* (Schwein.) Underw. due to the color of the ascomata; however, this species presents granulations along the stipe and lacks the green tones at the base, with ascospores of similar size, although slightly smaller (18–) 20–36 (–40) × 4–6 µm, as well as smaller asci 100–135 × 9–12 µm [37]. It can be separated from *M. fumosum* (Peck) E.J. Durand by the size of the ascospores; the spores of the latter species are broader (16–) 20–40 (–48) × 4–5 µm, and the ascomata are pale yellow, cinnamon brown to reddish ocher [37]. It is distinguished from *M. longisporum* E.J. Durand by its cinnamon brown ascomata and larger ascospores 40–90 (–100) × 4–6 µm. Macroscopically, it resembles *M. cyanobasis* P. Iglesias & Arauzo due to the green color at the base of the ascoma; however, ascomata are brown and not yellow as in *M. flavoviride*, where the ascospores are smaller, 15.4–22.5 × 4.4–6.1 µm, and paraphyses present different forms [38]. Likewise, it has similar shades at the base to *M. viride* (Schrad. ex J.F. Gmel.) Gillet; however, ascospores of the latter species differ in size and shape, measuring (11–) 18–22 (–25) × (4–) 5–7 µm, and are elliptical to oblong, sometimes curved, and without visible septa [39].

#### 3.1.4. Leotiomycetes, Helotiales, Helotiaceae

4. ***Claussenomyces paulinae*** Raymundo

**MycoBank:** MB842044

**Figure 7:**
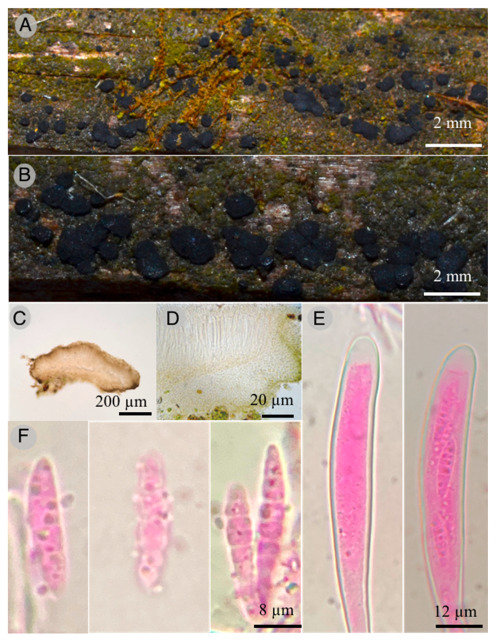
*Claussenomyces paulinae* T. Raymundo 7564 Holotype: (**A**,**B**) apothecia; (**C**,**D**) optical microscope images of apothecium; (**E**) optical microscope images of immature asci and mature asci with ascospores; (**F**) optical microscope images of ascospores with germination of conidia.

**Figure 8:**
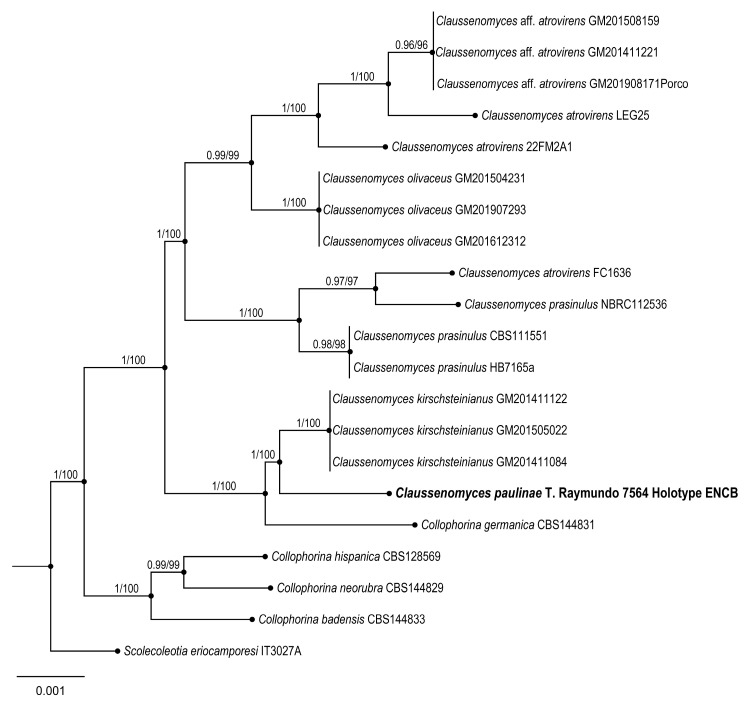
Bayesian inference phylogram of ITS sequence data. Posterior probability (left of slash) from Bayesian analysis and bootstrap support (right of slash) are given above the nodes. New species *Claussenomyces paulinae* is shown in bold.

**Figures:** Figure 7 and Figure 8.

**Diagnosis:** Apothecia 600–800 μm diameter, discoid to flat, pulvinate, dark, gelatinous consistency; asci 85–120 × 8–10 μm, claviform, septum simple at the base, second basal cell presents crosier; ascospores 18–22 × 3–3.5 μm, fusoid, with three septa and smooth wall, curved, hyaline, some germinating and forming conidia 4 × 2 μm.

**Type:** MEXICO. Hidalgo: Zacualtipán de Ángeles municipality, El Hayal forest, 20°37′41.6″ N, 98°36′58.4″ W, 2000 m, 30 May 2018, T. Raymundo 7577 (ENCB).

**GenBank:** ITS: OQ877256.

**Etymology:** Dedicated to Rosa Paulina Calvillo Medina for her contributions to Mexican mycology.

**Diagnosis**: Apothecia 600–800 μm in diameter and 600–800 μm in height, flat pulvinate to discoid, bright black color, substipitate, with gelatinous consistency, slightly verrucose texture; ectal excipulum epidermoid to globular, with cells 14–20 μm in diameter, hyaline to pale yellow and green at the margin, thin and smooth walls. Intricate medullar excipulum with swollen hyphae 2 μm in diameter, tapering toward the margin; hymenium hyaline 110 μm thick, filiform paraphyses with capitate apices; asci 85–120 × 8–10 μm, claviform with blunt apices and simple septum at the base; second basal cell presents crosier, biseriate apically when young and uniseriate when mature, obliquely located, octosporic, hyaline; ascospores 18–22 × 3–3.5 μm, fusoid with seven septa and smooth walls, curved, hyaline, some germinating and forming conidia 4 × 2 μm, ovoid, hyaline.

**Habitat**: Saprotrophic species found on decaying wood of *Pinus patula* Schiede ex Schltdl. & Cham.

**Additional specimens**: MEXICO: Hidalgo, Zacualtipán de Ángeles municipality, El Hayal forest, 20°37′41.6″ N, 98°36′58.4″ W, 2000 m, 30 May 2018, R. Valenzuela 18282 (ENCB).

**Taxonomical notes:** This species has dark gregarious apothecia with jelly consistency, inamyloid asci, and ascospore fragments form secondary spores. Morphologically, it is similar to *C. atrovirens* (Pers.) Korf & Abawi, which differs by forming dark green apothecia and ascospores with 4–7 septate [40,41]. Another similar species is *C. prassinulus* (P. Karst.) Korf & Abawi, which has emerald green apothecia with ascospores 13–14 × 3–3.5 μm [42,43]. Phylogenetically, *C. paulinae* is confirmed as an independent lineage forming an independent branch.

#### 3.1.5. Pezizomycetes, Pezizales, Chorioactidaceae

5. ***Wolfina molangoensis*** R. Valenz. & Raymundo

**MycoBank:** MB842045

**Figure 9:**
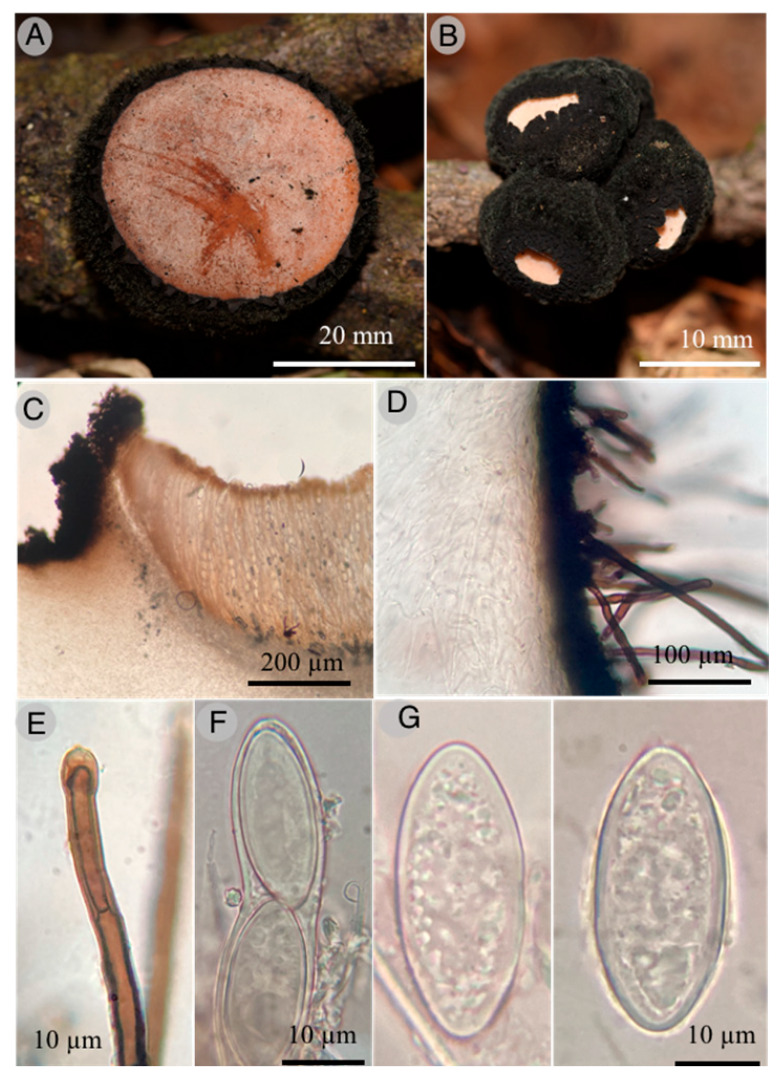
*Wolfina molangoensis* R. Valenzuela 18918 Holotype: (**A**) detail of apothecium; (**B**) apothecia; (**C**) optical microscope images of hymenium; (**D**) optical microscope images of ectal excipulum with hairs; (**E**) optical microscope images of detail of external hair; (**F**) optical microscope images of asci with ascospores; (**G**) optical microscope images of ascospores.

**Figure 10:**
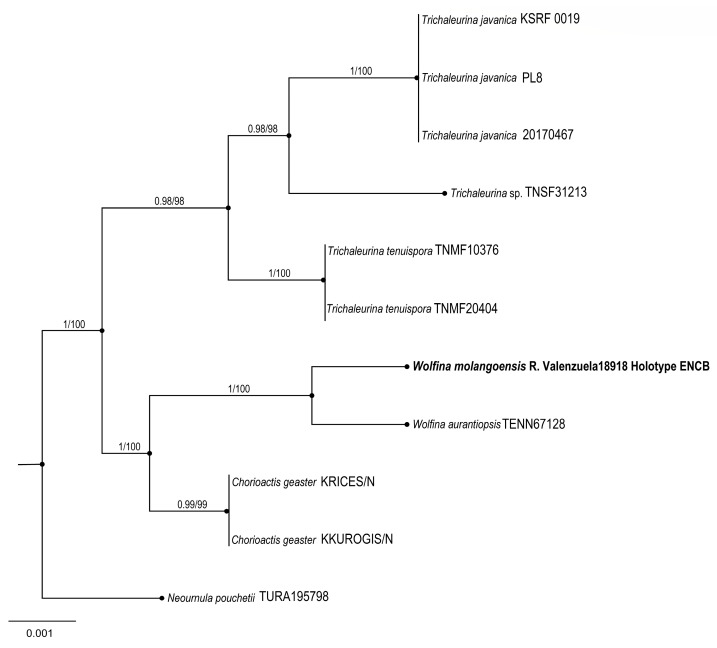
Bayesian inference phylogram of ITS sequence data. Posterior probability (left of slash) from Bayesian analysis and bootstrap support (right of slash) given above the node. New species *Wolfina molangoensis* is shown in bold. Boldface names indicate samples sequenced for this study.

**Figures:** Figure 9 and Figure 10.

**Diagnosis**: Apothecia 20–60 mm diameter, cup-shaped to discoid, external surface black, external hairs velvety; asci 400–450 × 22–24 μm, cylindrical, operculate; ascospores 35–40 × 14–18 μm, elliptical to cylindrical, hyaline, with granular content, smooth, thick-walled.

**Type**: MEXICO: Hidalgo. Molango municipality, Laguna de Atezca, 20°48′32″ N, 98°44′52″ W, alt. 1281 m, 31 May 2018, R. Valenzuela 18918 (ENCB, Holotype).

**GenBank:** ITS: OQ877257

**Etymology**: The name refers to the Molango locality in Hidalgo state.

Apothecia cup-shaped to discoid, sessile, 20–60 mm in diameter; hymenium shallow, pale orange (6A5) to peach (7A4), external surface black, velvety, convoluted, flesh thick, firm, corky when dried; external hairs cylindrical, 4–8 μm diameter, septate, walls up to 1 μm thick, brown, entire, smooth with apex lanceolate; ectal excipulum with pseudoparenchymatous texture, epidermoid cells with thick wall, dark brown; medullar excipulum intricate texture, hyphae hyaline, simple septate, 2–4 μm, wide hyphae; subhymenium of thick texture intricata, septate, 3–4 μm wide hyphae, arranged perpendicular to the asci; paraphyses 3–4 μm diameter, filiform, septate, anastomosing; asci 400–450 × 22–24 μm, cylindrical, operculate, with acute apex, walls up to 2 μm thick, octosporic, hyaline and inamyloid, tapering base and flexuous; ascospores 35–40 × 14–18 μm, elliptical to cylindrical, hyaline with granular content, sharp ends, thick-walled and smooth.

**Habitat:** Grows on branches of angiosperms.

**Additional specimens examined:** MEXICO: Hidalgo, Laguna de Atezca, 20°48′32″ N, 98°44′52″ W, 1281 m, 1 June 2018, T. Raymundo 7640 (ENCB, Paratype).

**Taxonomical notes**: Morphological and phylogenetically, this new species is close to *W. aurantiopsis* (Ellis) Seaver ex Eckblad; however, *W. aurantiopsis* forms apothecia 25–45 mm with yellow to ochraceous hymenium and ascospores 25–32 × 10–15 μm, elliptical to cylindrical, hyaline with granular content, rounded ends with thin-walled and striate. Argnello et al. [26] noted that it might be restricted to the eastern USA. We found differences in the size and form of spores between species.

#### 3.1.6. Sordariomycetes, Xylariales, Xylariaceae

6. ***Dematophora oaxacana*** Sánchez-Flores, R. Valenz. & Raymundo sp. nov.

**MycoBank:** MB842051.

**Figure 11:**
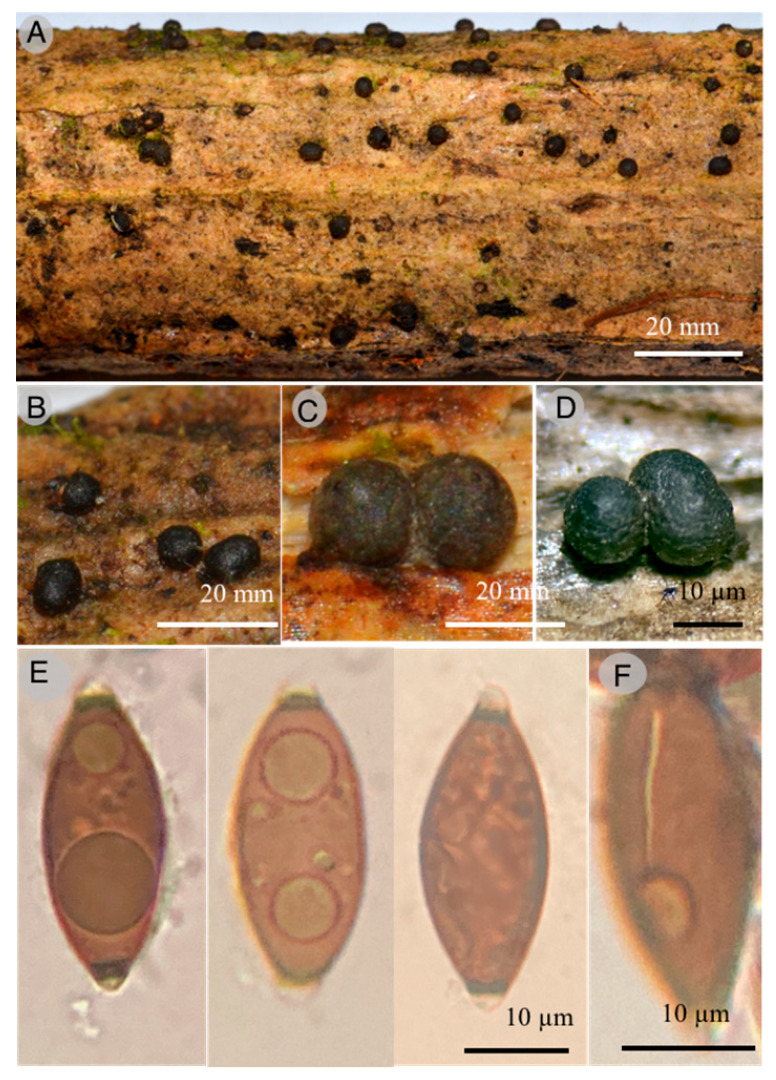
*Dematophora oaxacana* T. Raymundo 6161 Holotype: (**A**) stromata; (**B**–**D**) detail of stromata surface; (**E**) optical microscope images of ascospores; (**F**) optical microscope images of ascospores showing germinal line.

**Figure 12:**
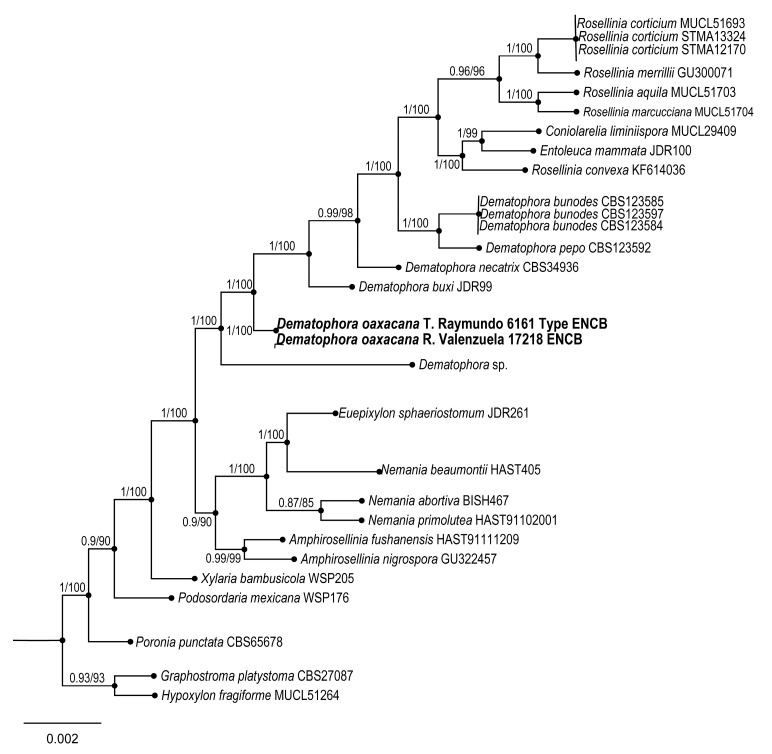
Bayesian inference phylogram of ITS, LSU, and *rpb*2 sequence data. Posterior probability (left of slash) from Bayesian analysis and bootstrap support (right of slash) given above the nodes. New species *Dematophora oaxacana* is shown in bold.

**Figures:**Figure 11 and Figure 12.

**Diagnosis:** Stromata 500–1100 × 400–700 µm, globose to subglobose, dark, solitary to gregarious, subiculum irregular extension, evanescent, carbonaceous, ascospores 20–29 × 10–13 (–14) µm, ovoid to asymmetrically ellipsoidal, with two cellular appendages, without germ slit.

**Type:** MEXICO: Oaxaca, Ixtlán de Juárez district, Santiago Comaltepec municipality, km 79 road Tuxtepec-Oaxaca, La Esperanza, Chinantla, 17°37′45″ N, 96°31′33″ W, 1130 m, 22 May 2017, T. Raymundo 6161 (ENCB, Holotype).

**GenBank:** ITS: OQ877258; nrLSU:OQ889487.

**Etymology**: The name refers to the state of Oaxaca, where this species was found.

**Diagnosis**: Stromata 500–1100 × 400–700 µm, globose to subglobose, dark to dark-brown, solitary, gregarious to cespitose, ostioles finely papillate to punctate; lack of subiculum; ectostroma dark, carbonaceous; endostroma 13–17 µm thick, light orange (5A4); perithecia not collapsed; asci dehiscent in 5% KOH; ascospores 20–29 × 10–13 (–14) µm, ovoid to asymmetrically ellipsoidal, brown, without germ slit, with flat sides ends, two cellular appendages, dehiscent in 5% KOH; external cellular appendage 3–5 µm tall and 5–6 µm wide, subglobose, hyaline; internal cellular appendage 1–2 µm tall and 2–3 µm wide, conical to subglobose, hyaline.

**Habitat:** Gregarious growing on decaying wood.

**Distribution:** Only known to be found in the state of Oaxaca.

**Additional specimens examined:** MEXICO, Oaxaca, Ixtlán de Juárez district, Santiago Comaltepec municipality, El Relámpago, La Esperanza, 17°35′28.1″ N, 96°53′52.2″ W, 1399 m, 29 May 2016, T. Raymundo 6161 (ENCB) and 6164 (ENCB). km 79 road Tuxtepec-Oaxaca, La Esperanza, Chinantla, 17°97′45″ N, 96°31′33″ W, 1130 m, 22 May 2017; 15 May 2015, R. Valenzuela 16111 (ENCB), 16145 (ENCB), T. Raymundo 5710 (ENCB). Loc. cit., 29 May 2016, R. Valenzuela 16667 (ENCB). Loc. cit., 22 May 2017, R. Valenzuela 17218 (ENCB), 17225 (ENCB), 17231 (ENCB), 17243 (ENCB), T. Raymundo 6587 (ENCB). Loc. cit., 23 May 2017, B. Nuñez 4 (ENCB), T. Raymundo 6607 (ENCB). Loc. cit., 30 April 2018, A. Cobos-Villagrán 1134 (ENCB). Paraje San Bernardo, La Esperanza, 17°37′55.4″ N, 96°22′1.5″ W, 25 September 2016, 1600 m, A. Trejo-Arana 17 (ENCB). Villa Alta district, Santiago Camotlán municipality, 5 km of Santiago Camotlán to San Juan Yatzuna, 24 March 2017, T. Raymundo (ENCB). Road Río Blanco, 25 March 2013, Galicia-Ávila 58 (ENCB). Santiago Camotlán, 25 March 2013, Escudero-Leyva 160 (ENCB).

**Taxonomical notes**: Ascospores measure 20–29.6 × 9.6–12 µm, without germline and double cell appendage. Phylogenetically, this species is close to *Dematophora buxi* (Fabre) C. Lamb., Wittstein & M. Stadler, differing from the latter in its macro and microscopic characteristics, as a more persistent subicula, with narrower ascospores 19.8–30.1 × 6–8.9 µm, fusoid, with straight germline and rounded apices. It is also similar to *D. francisiae* (L.E. Petrini) C. Lamb., Wittstein & M. Stadler; however, the latter has a persistent and felted subicula, 29–35 × 8–13 µm, longer ascospores, with a straight germline and rounded apices. Some species of *Dematophora* were earlier considered under the genus *Rosellinia* [44].

**Figure 13:**
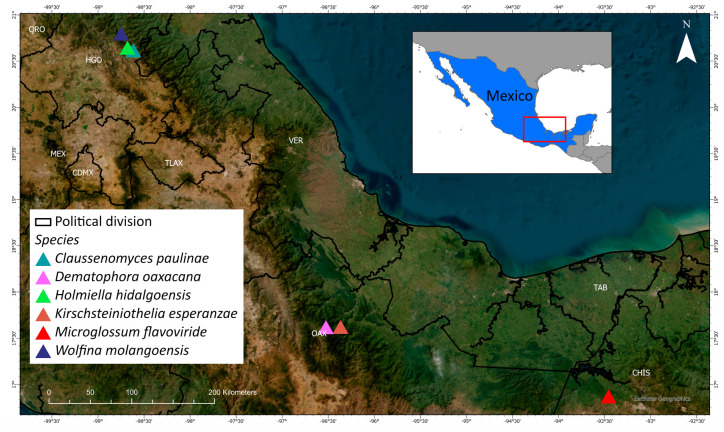
Distribution of new species.

The distribution of the described new species is shown in Figure 13.

## 4. Conclusions

**Figure 14:**
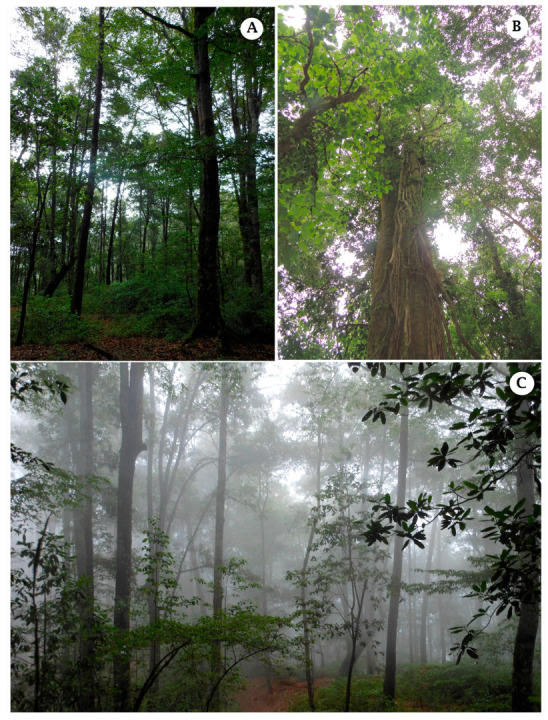
(**A**) View of *Fagus* tree in Mexican TMCF from Zacualtipán, Hidalgo. (**B**) View of *Oreomunnea mexicana* in La Esperanza, Oaxaca. (**C**) Tree components of Mexican TMCF in Zacualtipán, Hidalgo.

The Mexican tropical montane cloud forest (Figure 14) is one of the most diverse ecosystems for fungi. However, databases of other organisms, e.g., plants [45] and birds [46], but not fungi, are available for this ecosystem type. Unfortunately, they have not been extensively studied because of the lack of specialists; so, their representation in herbaria is poor. This study phylogenetically and morphologically describes six new species found in the Mexican TMCF.

Characterizing fungal diversity in TMCFs is relevant for forest conservation. These forests provide environmental services such as terrestrial biomass and water degradation and are the source of bioactive secondary metabolites [9].

In 2017, Del Olmo et al. [9] reported 954 Ascomycota species from the Mexican TMCF, and other recent studies added different species to the Mexican TMCF mycobiota. For example, Raymundo et al. [47] described *Marthamyces coronadoae,* Raymundo et al. [48] described seven species of Hypocreales, Arias et al. [49] registered the asexual phases of 355 species, Medel-Ortiz et al. [50] found seven new records for the TMCF, and Raymundo et al. [41] recorded 10 new species in Mexico. Other studies that recorded new taxa are as follows (in chronological order): Sánchez-Flores et al. [51] described *Hymenoscyphus herrerae* from Puebla and registered six new species in the country; Raymundo et al. [43] recorded 17 new species from different TMCF localities; and Cobos-Villagrán et al. [52] registered *Rhytidhysteron esperanzae* and *R. mesophila* from Oaxaca and Hidalgo, respectively. In Puebla, three studies are relevant: Barbosa-Reséndiz et al. [53] described *Daldinia rehmii*, Raymundo et al. [54] recorded *Unguiculariopsis ravenelii*, and Sánchez-Flores et al. [55] described *Ionomidotis mesophile*. Then, Raymundo et al. [56] described *Smardaea isoldae* from Hidalgo, and Valenzuela et al. [57] added 10 new records for the TMCF in Oaxaca. In Veracruz, Chacón-Zapata and Gonzalez [58] described *Euacanthe renispora*, Guzmán-Guillermo et al. [59] described *Paruephaedria heimerlii*, and Chacón-Zapata and Ramirez-Guillén [60] listed 11 new records of Coronophorales. Finally, de la Fuente et al. [61] described *Elaphomyces castilloi* from Chiapas. The above information allows us to assume the existence of at least 1389 species inhabiting the Mexican TMCF. As González et al. [5] suggested, the precise number of species is difficult to establish due to nomenclature changes and the imprecision of Ascomycetes species identification.

Among the six new species described in this study, three species are distributed in Hidalgo, Sierra Madre Oriental, a mountainous area characterized by its abrupt topography and high beta diversity. Two were re-collected in Sierra de Juárez (Sierra Norte de Oaxaca), and one in Lagunas de Montebello, Altos de Chiapas, on the southern border with Guatemala. It is worth mentioning that the genera *Holmiella* and *Wolfina* are cited for the first time in the country.

Mexico is one of the world’s most diverse areas for fungi; so, it is essential to inventory and describe the fungal species in this type of ecosystem. TMCFs are the most threatened terrestrial ecosystems at the national level and are classified as “habitats in danger of extinction” [62]. In addition, a meta-analysis recently revealed that Mexico is a hotspot for oak species and their ectomycorrhizal mycobionts [63]. Those authors considered that the Mexican oak forests are essential for maintaining biodiversity due to the richness and endemism of fungi, mainly those associated with Fagaceae.

The loss of the TMCF is due to its transformation into grazing land for livestock and agriculture, mainly for avocados and coffee. The fungal abundance is strongly affected by the loss of this ecosystem type. The effects of global warming have not yet been evaluated in the case of these fungi.

## Figures and Tables

**Table 1:** GenBank accession numbers corresponding to sequences used in phylogenetic analyses of *Holmiella hidalgoensis* sp. nov. Accessions of new species indicated in bold.

Species Name	Isolate/Voucher/Strain	GenBank Accessions		
		ITS	nrLSU	SSU
*Anisomeridium ubianum* (Vain.) R.C. Harris	MPN94	KY486750	GU327709	JN887379
*Cryomyces antarcticus* Selbmann, de Hoog, Mazzaglia, Friedman & Onofri	CCFEE 536	-----	GU250365	GU250321
*Cryomyces minteri* Selbmann, de Hoog, Mazzaglia, Friedman & Onofri	CCFEE 5187	-----	KC315869	KC315858
*Glyphium elatum* (Grev.) H. Zogg	EB 0388	KM220946	KM220940	-----
	EB 0342	KM220945	KM220938	KM220935
	EB 0329	-----	KM220937	KM220934
	EB 0365	-----	KM220939	KM220936
*Glyphium grisonense* Math.	EB 0376	-----	KM220942	-----
***Holmiella hidalgoensis***	**T. Raymundo 4608 Holotype ENCB**	**OQ877252**	**OQ880481**	**OQ878242**
*Holmiella junipericola* Pem, Gafforov, Jeewon & K.D. Hyde	MFLUCC 18-0503	MH188902	MH188900	MH188901
*Holmiella juniperi-semiglobosae* Pem, Gafforov, Jeewon & K.D. Hyde	MFLUCC 17-1955	MH188905	MH188903	MH188904
*Holmiella Sabina* (De Not.) Petrini, Samuels & E. Müll.	G.M. 2015-04-29.2	KY486750	-----	-----
*Hysteropatella clavispora* (Peck.) Hönh.	CBS 247.34	-----	AY541493	AY511483
*Hysteropatella elliptica* (Fr.) Rehm	G.M. 2013-05-06 01	-----	KM220948	KM220948
	CBS 935.97	-----	DQ767657	EF495114
*Hysteropatella prostii* (Duby) Rehm	H.B. 9934b	KT876980	KT876980	-----
	G.M. 2014-05-20 01	-----	KM220949	-----
*Lichenothelia calcarea* Henssen	L1324	-----	KC015062	KR045803
*Lichenothelia convexa* Henssen	L1609	-----	KC015071	KR045805
*Patellaria atrata* (Hedw.) Fr.	BCC 28877	KM220950	GU371829	-----
	BCC 28876	-----	KM220950	-----
	CBS 958.97	-----	GU301855	-----
*Yuccamyces citri* Crous	CBS 143161	MG386043	MG386096	
*Yuccamyces pilosus* (R.F. Castañeda) R.F. Castañeda	CBS 579.92	-----	MG386097	-----

**Table 2:** GenBank accession numbers corresponding to sequences used in phylogenetic analyses of *Kirschsteiniothelia esperanzae* sp. nov. Accessions of new species indicated in bold.

Species Name	Isolate/Voucher/strain	GenBank Accessions	
		ITS	nLSU
*Acrospermum adeanum* Höhn.	M133	EU940180	EU940104
*Acrospermum compressum* Tode	M151	EU940161	EU940084
*Acrospermum graminum* Lib.	M152	EU940162	EU940085
*Kirschsteiniothelia aethiops* (Sacc.) D. Hawksw.	CBS 109.53	-----	AY016361
	MFLUCC 16–1104	MH182583	MH182589
	S–783	MH182586	MH182595
	MFLUCC 15–0424	KU500571	KU500578
*Kirschsteiniothelia aquatica* Z.L. Luo, K.D. Hyde & H.Y. Su	MFLUCC 17–1685	MH182587	MH182594
*Kirschsteiniothelia arasbaranica* Mehrabi, Hemmati & Asgari	IRAN 2509C	KX621986	KX621987
	IRAN 2508C	KX621983	KX621984
*Kirschsteiniothelia cangshanensis* Z.L. Luo, D.F. Bao, K.D. Hyde & H.Y. Su	MFLUCC 16–1350	MH182584	MH182592
***Kirschsteiniothelia esperanzae***	**T. Raymundo 6581 Holotype ENCB**	**OQ877253**	**OQ880482**
*Kirschsteiniothelia fluminicola* Z.L. Luo, K.D. Hyde & H.Y. Su	MFLUCC 16–1263	MH182582	MH182588
*Kirschsteiniothelia lignicola* Boonmee & K.D. Hyde	MFLUCC 10–0036	HQ441567	HQ441568
*Kirschsteiniothelia nabanheensis* Jing W. Liu & Jian Ma	HJAUP C2006	OQ023274	OQ023275
	HJAUP C2004	OQ023197	OQ023273
*Kirschsteiniothelia phoenicis* S. N. Zhang & K.D. Hyde	MFLUCC 18–0216	MG859978	MG860484
*Kirschsteiniothelia rostrata* Jing Yang & K.D. Hyde	MFLUCC 15–0619	KY697280	KY697276
	MFLUCC 16–1124	-----	MH182590
*Kirschsteiniothelia submersa* Hong Y. Su & K.D. Hyde	MFLUCC 15–0427	KU500570	KU500577
	S–481	-----	MH182591
	S–601	MH182585	MH182593
*Kirschsteiniothelia tectonae* Doilom, Bhat & K.D. Hyde	MFLUCC 12–0050	KU144916	KU764707
*Kirschsteiniothelia thailandica* Y.R. Su, Yong Wang bis & K.D. Hyde	MFLUCC 20–0116	MT985633	MT984443
*Kirschsteiniothelia thujina* (Peck.) D. Hawksw.	JF 13210	KM982716	KM982718
*Megalotremis verrucosa* (Makhija & Patw.) Aptroot	MPN104	-----	GU327718
*Phyllobathelium anomalum* Lücking	MPN 242	-----	GU327722
*Phyllobathelium firmum* (Stirt.) Věsda	ERP 3175	-----	GU327723
*Pseudorobillarda eucalypti* Tangthir. & K.D. Hyde	MFLUCC 12–0422	KF827451	KF827457
*Pseudorobillarda phragmitis* (Cunnell) M. Morelet	CBS 398.61	MH858101	EU754203
*Strigula guangxiensis* S.H. Jiang, X.L. Wei & J.C. Wei	HMAS-L0138040	NR146255	MK206256
*Strigula macrocarpa* Vain.	HMAS-L0141394	-----	MK206240
*Strigula nemathora* Mont.	MPN 72	-----	JN887405
*Strigula nitidula* Mont.	HMAS-L0139358	-----	MN788374
*Strigula sinoaustralis* S.H. Jiang, X.L. Wei & J.C. Wei	HMAS-L0137204	-----	MK206249
*Strigula univelbiserialis* S.H. Jiang, X.L. Wei & J.C. Wei	HMAS-L0137657	-----	MK206243
*Tenuitholiascus porinoides* S.H. Jiang & J.C. Wei	HMAS-L0139638	-----	MK206259
	HMAS-L0139639	-----	MK206258
	HMAS-L0139640	-----	MK206260

**Table 3:** GenBank accession numbers corresponding to sequences used in phylogenetic analyses of *Microglossum flavoviride* sp. nov. Accessions of new species indicated in bold.

Species Name	Isolate/Voucher/Strain	GenBank Accessions		
		ITS	nLSU	*rpb2*
*Microglossum clavatum* V. Kučera, Lizoň & Tomšovský	SAV F-11276	KX382864	KX382864	KX382884
	SAV F-11272	KX382841	-----	-----
	SAV F-11074	KX382865	KX382865	KX382885
*Microglossum cyanobasis* P. Iglesias & Arauzo	AH 43985	KX371850	-----	-----
***Microglossum flavoviride***	**García 18649 Holotype ITCV**	**OQ877254**	**OQ880483**	
	**García 18686**	**OQ877255**	**OQ880484**	
*Microglossum fuscorubens* Boud.	ERRO 2012120704	KX371856	-----	-----
	ERRO 2012120705	KX371857	-----	-----
	ERRO 2012120706	KX371858	-----	-----
	SAV F-11275	KX382834	KX382834	KX382883
*Microglossum griseoviride* V. Kučera, Lizoň & Tomšovský	SAV F-9920	KX595249	KC595250	KX382872
	SAV F-10699	KC595261	-----	-----
	SAV F-10696	KX382857	-----	-----
*Microglossum nudipes* Boud.	SAV F-11053	KX382838	KX382867	-----
	SAV F-11051	KX382856	-----	-----
	SAV F-11274	KX382836	KX382836	KX382888
	SAV F-11285	KX382859	KX382869	KX382887
	SAV F-11271	KX382837	-----	-----
*Microglossum olivaceum* (Pers.) Gillet	KM135962	EU784374	-----	-----
	KM135599	EU784373	-----	-----
	ERRO 2004110702	KX371853	-----	-----
*Microglossum parvisporum* V. Kučera, Lizoň & Tomšovský	SAV F-10998	KM114901	KM114901	KX382879
	LE 291852	KX382839	-----	-----
	SAV F-11283	KM114901	KM114901	KX382879
*Microglossum pratense* V. Kučera, Tomšovský & Lisoš	SAV F-10024	KC595259	KC595260	KX382880
	SAV F-11020	KJ513006	KJ513006	KX382881
	O 64797	KJ513004	-----	-----
	O 294564	KJ513005	-----	-----
	O 170878	KJ513002	-----	-----
	O 270070	KJ513003	-----	-----
	SAV F-11062	KX382848	-----	-----
	LE 294492	KX382849	-----	-----
	LE 294489	KX382850	-----	-----
	SAV F-10568	KX382851	-----	-----
	SAV F-11056	KX382847	-----	-----
	SAV F-11052	KX382852	-----	-----
*Microglossum rufescens* (Grelet) Bon	SAV F-9921	KC595257	-----	-----
	ERRO 2004110703	KX371854	-----	-----
	ERRO 2011122601	KX371855	-----	-----
	SAV F-11282	KX382858	KX382868	KX382892
	SAV F-11204	KX382835	KX382866	KX382893
*Microglossum rufum* (Schwein.) Underw.	Ingo 163	DQ257360	-----	-----
*Microglossum tenebrosum* V. Kučera, Tomšovský, Lisoš & F. Hampe	SAV F-11273	KX382842	-----	-----
	SAV F-11278	KX382845	KX382845	KX382891
	SAV F-11279	KX382843	-----	-----
	SAV F-11070	KX382846	KX382846	KX382890
	SAV F-11072	KX382844	KX382844	KX382889
*Microglossum truncatum* V. Kučera, Tomšovský & Lisoš	SAV F-11023	KJ513009	KJ513009	KX382874
	SAV F-10720	KX382840	-----	-----
	O 224247	KJ513010	-----	-----
	SAV F-11280	KX382861	KX382861	KX382875
	SAV F-11022	KJ513011	-----	-----
	SAV F-11064	KX382855	-----	-----
	LE 291847	KX382863	KX382871	KX382876
	SAV F-11262	KX382862	KX382862	KX382877
	SAV F-11261	KX382853	-----	-----
	SAV F-11263	KX382854	-----	-----
*Microglossum viride* (Schrad. ex J.F. Gmel.) Gillet	SAV F-10249	KC595253	KC595254	KX382873
	SAV F-10697	KC595265	-----	-----
	SAV F-10698	KC595263	-----	-----
	KM90199	EU784375	-----	-----

**Table 4:** GenBank accession numbers corresponding to sequences used in phylogenetic analyses of *Claussenomyces paulinae* sp. nov. Accessions of new species indicated in bold.

Species Name	Isolate/Voucher/Strain	GenBank Accessions
		ITS
*Claussenomyces atrovirens* (Pers.) Korf & Abawi	22FM2A1	MW709917
*Claussenomyces atrovirens* (Pers.) Korf & Abawi	LEG25	MW204926
*Claussenomyces atrovirens* (Pers.) Korf & Abawi	FC1636	LC425048
*Claussenomyces* aff. *Atrovirens*	GM20144422.1	MW178207
*Claussenomyces* aff. *Atrovirens*	GM20150815.9	MT949706
*Claussenomyces* aff. *Atrovirens*	GM20190817.1	MT522872
*Claussenomyces kirschsteinianus* (Kirschst.) G. Marson & Baral	GM20150502.2	KY689631
*Claussenomyces kirschsteinianus* (Kirschst.) G. Marson & Baral	GM20141112.2	KY689629
*Claussenomyces kirschsteinianus* (Kirschst.) G. Marson & Baral	GM20141108.4	KY689628
*Claussenomyces olivaceus* (Fuckel) Sherwood	GM20150423.1	KY661433
*Claussenomyces olivaceus* (Fuckel) Sherwood	GM20190729.3	OP103955
*Claussenomyces olivaceus* (Fuckel) Sherwood	GM20161231.2	MW167780
***Claussenomyces paulinae***	**T. Raymundo 7564 Holotype ENCB**	**OQ877256**
*Claussenomyces prasinulus* (P. Karst.) Korf & Abawi	HB7165a	OM808929
	CBS111551	MN082653
	NBRC 112536	LC488725
*Collophorina badensis* S. Bien & Damm	CBS144833	NR165902
*Collophorina germanica* S. Bien & Damm	CBS144831	NR165903
*Collophorina hispanica* (Gramaje, Armengol & Damm) Damm & Crous	CBS128569	MH864962
*Collophorina neorubra* S. Bien & Damm	CBS144829	NR165901
*Scolecoleotia eriocamporesi* H. B. Jiang, Phookamsak & K.D. Hyde	IT3027A	MW981448
*Scolecoleotia eriocamporesi* H. B. Jiang, Phookamsak & K.D. Hyde	IT3027B	MW981449

**Table 5:** GenBank accession numbers corresponding to sequences used in phylogenetic analyses of *Wolfina molangoensis* sp. nov. Accessions of new species indicated in bold.

Species Name	Isolate/Voucher/Strain	GenBank Accessions
		ITS
*Chorioactis geaster* (Peck) Kupfer ex Eckblad	K. Rice s.n.	AY307936
	S. Kurogi s.n.	AY307937
*Trichaleurina javanica* (Peck) M. Carbone, Agnello & P. Alvarado	KSRF 0019	MF476196
	PL8	MZ061709
	20170467	MK184529
*Trichaleurina* sp.	TNS-F-31213	KF418250
*Trichaleurina tenuispora* M. Carbone, Yei Z. Wang & Cheng L. Huang	TNM F10376	NR159000
	TNM F20404	KF418249
*Wolfina aurantiopsis* (Ellis) Seaver ex Eckblad	TENN 67128	KC306744
***Wolfina molangoensis***	**R. Valenzuela 18918 Holotype ENCB**	**OQ877257**
*Neournula pouchetii* (Berthet & Riousset) Paden	TURA195798	JX669837

**Table 6:** GenBank accession numbers corresponding to sequences used in phylogenetic analyses of *Dematophora oaxacana* sp. nov. Accessions of new species indicated in bold.

Species Name	Isolate/Voucher/Strain	GenBank Accessions	
		ITS	nLSU
*Amphirosellinia fushanensis* Y.M. Ju, J.D. Rogers & H.M. Hsieh	HAST 91111209	GU339496	-----
*Amphirosellinia nigrospora* Y.M. Ju, J.D. Rogers & H.M. Hsieh	HAST 91092308	GU322457	-----
*Coniolarelia limoniispora*	MUCL 29409	MN984615	MN984624
*Dematophora bunodes* (Berk. & Broome) C. Lamb., Wittstein & M. Stadler	CBS 123584	MN984617	-----
*Dematophora bunodes* (Berk. & Broome) C. Lamb., Wittstein & M. Stadler	CBS 123585	MN984618	-----
*Dematophora bunodes* (Berk. & Broome) C. Lamb., Wittstein & M. Stadler	CBS 123597	MN984619	MN984625
*Dematophora buxi* (Fabre) C. Lamb., Wittstein & M. Stadler	JDR 99	GU300070	-----
*Dematophora necatrix* R. Hartig	CBS 349.36	AY909001	KF719204
*Dematophora necatrix* R. Hartig	W 97	DF977487	DF977487
***Dematophora oaxacana***	**T. Raymundo 6161 Holotype ENCB**	**OQ877258**	**OQ880487**
*Dematophora oaxacana* Sánchez-Flores, R. Valenz. & Raymundo	R. Valenzuela 17218 ENCB	OQ877259	OQ880488
*Dematophora pepo* (Pat.) C. Lamb., Wittstein & M. Stadler	CBS 123592	MN984620	-----
*Entoleuca mammata* (Wahlenb.) J.D. Rogers & Y.M. Ju	JDR 100	GU300072	-----
*Euepixylon sphaeriostomum* (Schwein.) Lar.N. Vassiljeva & S.L. Stephenson	JDR 261	GU292821	-----
*Graphostroma platystomum* (Schwein.) Piroz	CBS 270.87	JX658535	DQ836906
*Hypoxylon fragiforme* (Pers.) J. Kickx f.	MUCL 51264	KC477229	KM186295
*Nemania abortiva* J.D. Rogers, Y.M. Ju & Hemmes	BISH 467	GU292816	-----
*Nemania beaumontii* (Berk. & M.A. Curtis) Y.M. Ju & J.D Rogers	HAST 405	GU292819	-----
*Nemania beaumontii* (Berk. & M.A. Curtis) Y.M. Ju & J.D Rogers	FL 0980	-----	JQ760608
*Nemania bipapillata* (Berk. & M.A. Curtis) Pouzar	HAST 90080610	GU292818	-----
*Podosordaria mexicana* Ellis & Holw.	WSP 176	GU324762	-----
*Podosordaria punctata*	CBS 656.78	KT281904	KY610496
*Rosellinia aquila* (Fr.) Ces. & De Not.	MUCL 51703	KY610392	KY610460
*Rosellinia marcucciana* Ces	MUCL 51704	MN984616	MN984626
*Rosellinia corticium* (Schwein.) Sacc.	MUCL 51693	KY610393	KY610461
	STMA 13324	MN984621	MN984627
	STMA 12170-15209	MN984623	MN984629
*Rosellinia nectrioides* Rehm	CBS 449.89	MN984622	MN984628
*Xylaria arbuscula* Sacc.	CBS 126415	KY610394	KY610463
*Xylaria hypoxylon* (L.) Grev.	CBS 122620	KY204024	KY610495
*Xylaria bambusicola* Y.M. Ju & J.D Rogers	WSP 205	EF026123	-----

## Data Availability

No new data were created or analyzed in this study. Data sharing is not applicable to this article.
